# Associations between social determinants of health and mental health disorders among U.S. population: a cross-sectional study

**DOI:** 10.1017/S2045796024000866

**Published:** 2025-01-15

**Authors:** S. Tanarsuwongkul, J. Liu, M. Spaulding, K. Perea-Schmittle, M. Lohman, Q. Wang

**Affiliations:** 1 Department of Chemistry and Biochemistry, University of South Carolina, Columbia, SC, USA; 2 Epidemiology and Biostatistics, Arnold School of Public Health, University of South Carolina, Columbia, SC, USA; 3 Department of Biomedical Engineering, Molinaroli College of Engineering and Computing, University of South Carolina, Columbia, SC, USA; 4 Department of Biology, New Mexico Institute of Mining and Technology, Socorro, NM, USA

**Keywords:** All of Us, anxiety disorder, major depression, SDOH, social determinants of health

## Abstract

**Aims:**

The impact of social determinants of health (SDOH) on mental health is increasingly realized. A comprehensive study examining the associations of SDOH with mental health disorders has yet to be accomplished. This study evaluated the associations between five domains of SDOH and the SDOH summary score and mental health disorders in the United States.

**Methods:**

We analyzed data from a diverse group of participants enrolled in the All of Us research programme, a research programme to gather data from one million people living in the United States, in a cross-sectional design. The primary exposure was SDOH based on Healthy People 2030: education access and quality, economic stability, healthcare access and quality, social and community context, and neighbourhood and built environment. A summary SDOH score was calculated by adding each adverse SDOH risk (any SDOH vs. no SDOH). Our primary outcomes were diagnoses of major depression (MD) (i.e., major depressive disorder, recurrent MD or MD in remission) and anxiety disorders (AD) (i.e., generalized AD and other anxiety-related disorders). Multiple logistic regression models were used to determine adjusted odd ratios (aORs) for MD and/or ADs after controlling for covariates.

**Results:**

A total of 63,162 participants with MD were identified (22,277 [35.3%] age 50–64 years old; 41,876 [66.3%] female). A total of 77,624 participants with AD were identified (25,268 [32.6%] age 50–64 years old; 52,224 [67.3%] female). Factors associated with greater odds of MD and AD included having less than a college degree, annual household income less than 200% of federal poverty level, housing concerns, lack of transportation, food insecurity, and unsafe neighbourhoods. Having no health insurance was associated with lower odds of both MD and AD (aOR, 0.48; 95% confidence interval [CI], 0.46–0.51 and aOR, 0.44; 95% CI, 0.42–0.47, respectively). SDOH summary score was strongly associated with the likelihood of having MD and AD (aOR, 1.97; 95% CI, 1.89–2.06 and aOR, 1.69; 95% CI, 1.63–1.75, respectively).

**Conclusions:**

This study found associations between all five domains of SDOH and the higher odds of having MD and/or AD. The strong correlations between the SDOH summary score and mental health disorders indicate a possible use of the summary score as a measure of risk of developing mental health disorders.

## Introduction

Mental health disorders are the leading cause of disability worldwide. In 2019, 970 million people across the globe were living with mental disorders. Anxiety and depressive disorders are the most prevalent disorders, affecting around 301 and 280 million people worldwide, respectively (World Health Organization, 2022). According to the Global Health Estimates 2019, depressive disorders and anxiety disorders (AD) are the second and sixth in the 20 leading causes of years lost due to disability and depressive disorders are one of the top 20 leading causes of disability-adjusted life year (DALY) (World Health Organization, 2020). Furthermore, it was estimated that mental health disorders resulted in $418 million DALY or an estimated $5 trillion in economic value (Arias *et al.*, 2022).

The COVID-19 pandemic led to a rise in the prevalence of mental health disorders (Gray *et al.*, 2022), which disproportionately impacted those with limited economic resources (Ettman *et al.*, 2020). The high prevalence of mental health disorders can be reduced by macro-policy interventions to lessen economic insecurity and improve housing, education and nutrition (Saxena *et al.*, 2006). There is growing recognition of the impact of various economic conditions on mental health outcomes. Extensive research has demonstrated that education, income and occupation are reliable predictors for a broad spectrum of mental health outcomes. Moreover, unemployment is consistently associated with increased psychological distress (Mao and Agyapong, 2021). These factors are known as social determinants of health (SDOH), which are the environmental factors that have an impact on a variety of risks and outcomes related to health, functioning and quality of life.

SDOH encompasses five core domains: economic stability, education access and quality, healthcare access and quality, neighbourhood and built environment, and social and community context (Department of Health and Human Services, 2020). The World Health Organization estimates that SDOH contributes to approximately one-third to one-half of the origins of health issues (Tasman, 2023). Population health can be significantly improved by addressing socio-economic factors that underpin health inequalities, according to Thomas Frieden’s Five-Tier Health Impact Pyramid (Thornton *et al.*, 2016). Thus, a comprehensive analysis of the associations between SDOH factors and health outcomes can provide invaluable insights for developing more effective health policies. This aligns with one of the overarching goals of Healthy People 2030, that is, to improve overall population health and reduce health disparities through addressing SDOH (Department of Health and Human Services, 2020). However, to our knowledge, no study has been conducted to fully examine how each of the five SDOH domains affects mental health individually and collectively (Mao and Agyapong, 2021).

In this work, we used the data from the All of Us research programme (Denny *et al.*, 2019) to examine the associations between all five domains of SDOH and two major mental health disorders: major depression (MD) and AD. A comprehensive SDOH summary score that incorporates data from all SDOH domains was used to evaluate associations between the collective effects of SDOH and mental health disorders.

## Methods

### Data source and study population

The All of Us research programme (www.allofus.nih.gov) is a research programme with the mission of advancing precision medicine research through the collection and open dissemination of health data from one million Americans (Denny *et al.*, 2019). Data in the All of Us research programme includes both information from electronic health records and self-report data from consented individuals aged 18 years old or older living in the United States. By 2022, 78% of participants in the All of Us research programme were drawn from historically under-represented groups in biomedical research (Ramirez *et al.*, 2022). Thus, data in the research programme is uniquely qualified to study the associations between SDOH and mental health disorders among Americans (Kirby and Slone, 2021). The protocol of the All of Us research programme can be found elsewhere (National Institutes of Health (NIH)—All of Us, 2021).

The release version number 7, which comprises data from 413,457 participants who enrolled between 30 May 2017 and 1 July 2022, was used. The All of Us protocol and associated materials were approved by the All of Us Institutional Review Board. This study analyzed the publicly available deidentified data, which is exempted from a review by the institutional review board.

### Mental health disorders

Participants with the condition of major depressive disorder, recurrent MD, or MD in remission were included in the MD group. The AD group included generalized AD and other anxiety-related disorders such as acute stress disorder, obsessive-compulsive disorder and post-traumatic stress disorders. We used the Observational Medical Outcomes Partnership codes and Systematized Nomenclature of Medicine (SNOMED) codes in the Condition Domain to identify mental health disorders from the electronic medical records (**Table S1**). This included participants in remission, where applicable, because many patients with these disorders continue to have residual impairment in functioning long after their symptoms have resolved (Sheehan *et al.*, 2011). For control groups, All of Us participants were randomly selected at a 1:1 ratio to the number of participants who have MD and ADs, after excluding participants with MD and AD based on the selection criteria mentioned above. df.sample() in Python was used to randomly select the control groups with random_state = 1 to ensure the reproducibility of the results.

### Social determinants of health measure

Using available data in the All of Us research programme, the five domains of SDOH were measured by the highest education attainment, annual household income, health insurance coverage, housing concern in the last 6 months, delayed care due to transportation, food insecurity level in the past 12 months and neighbourhood safety in our study (see **Supplementary information**). Each adverse SDOH risk factor was assigned a score of one to participants who did not complete high school education, were from a household earning <200% of the federal poverty level (ASPE, 2023), reported housing concerns within the previous 6 months, did not have health insurance, experienced transportation-related delays in receiving care, reported food insecurity within the previous year or lived in unsafe neighbourhood. A score of 0.5 was allocated to individuals who had completed high school, but not college. Those who did not provide any SDOH data were placed into the ‘Did not answer’ category and retained in the analyses. A summary score was calculated by adding each adverse SDOH risk. A score of zero indicated no SDOH risk, while a score of seven indicated the highest SDOH score. Participants were categorized into three groups: those with SDOH risk (score > 0), those without any SDOH risk (score = 0) and those with incomplete information (missing any SDOH items and did not have any risk, if answered). The scoring system is shown in **Table S2**.

### Covariates

Based on previous studies about the correlates of mental health disorders (Bailey *et al.*, 2019; Gao *et al.*, 2023; Hallers-Haalboom *et al.*, 2020; Jones *et al.*, 2014; Maclntyre *et al.*, 2023; Mitina *et al.*, 2020; Yan *et al.*, 2021; Zhao *et al.*, 2020), the following covariates were considered in our study: race/ethnicity (Hispanic/Latino/a/x, non-Hispanic White, non-Hispanic Black, non-Hispanic Asian, non-Hispanic others and non-Hispanic > 1 races), age at consent (18–34, 35–49, 50–64, and 65 and above), sexual orientation (female, male, and LGBTQIA+), and any disabilities (blind, deaf, difficulty dressing/bathing, difficulty running errands alone, difficulty concentrating and difficulty walking/climbing).

### Statistical analyses

In our cross-sectional study, we estimated the prevalences of MD and AD overall and for each subtype of covariates and SDOH. Multiple logistic regression models were used to estimate the odds of having MD and AD for each SDOH indicator and SDOH summary score while adjusting for race/ethnicity, sexual orientation, age, and disabilities. Crude and adjusted odd ratios [aOR] and the 95% confidence intervals (CI) of MD and AD were presented. A two-tailed *P*-value of <0.05 was considered to be statistically significant. Analyses were carried out using Python3 in a Jupyter Notebook environment. The logit() function from the statsmodels module was used. The analyses were conducted from May 2023 to September 2023.

## Results

### All of Us participant characteristics

Data from 413,457 participants were included in the All of Us research programme as of 1 July 2022. Of these, 247,453 (59.8%) were women, 222,646 (53.8%) were non-Hispanic White and 77,069 (18.6%) were non-Hispanic Black. The age was 50.65 ± 16.21 (mean ± SD) years old.

Overall, 36,432 participants (8.8%) received education below high school and 139,389 participants (33.7%) had an annual household income <200% of the federal poverty level. Furthermore, 26,779 participants (6.5%) had no health coverage, and 9,642 participants (2.3%) felt that their neighbourhoods were unsafe. In the last 6 months before the survey, 66,649 participants (16.1%) had housing concerns. In the last 12 months before the survey, 14,129 participants (3.4%) had delayed getting the medical care they needed due to transportation. Moreover, 3,548 participants (0.9%) had food insecurity (Table 1).

**Table 1.** Characteristics of All of Us participants and prevalences of major depression and anxiety disorder

Characteristics	All of Us participants *n* (%)	Major depression *n* (%)	Anxiety disorder *n* (%)
**Total**	413 457 (100.0)	63 162 (15.3)	77 624 (18.8)
**Race/Ethnicity**			
Hispanic/Latino/a/x	74 114 (17.9)	11 029 (14.9)	13 264 (17.9)
Non-Hispanic White	222 646 (53.8)	35 847 (16.1)	46 479 (20.9)
Non-Hispanic Black	77 069 (18.6)	11 487 (14.9)	11 764 (15.3)
Non-Hispanic Asian	13 838 (3.3)	776 (5.6)	1 125 (8.1)
Non-Hispanic, >1 races	6 836 (1.7)	1 030 (15.1)	1 302 (19.0)
Other	7 262 (1.8)	1 091 (15.0)	1 398 (19.3)
Did not answer	11 692 (2.8)	1 902 (16.3)	2 292 (19.6)
**Age at consent**			
18–34 y	99 133 (24.0)	11 740 (11.8)	16 674 (16.8)
35–49 y	94 319 (22.8)	15 046 (16.0)	19 381 (20.5)
50–64 y	126 638 (30.6)	22 277 (17.6)	25 268 (20.0)
≥ 65 y	93 365 (22.6)	14 099 (15.1)	16 301 (17.5)
**Sexual orientation**			
Female	247 453 (59.8)	41 876 (16.9)	52 224 (21.1)
Male	154 241 (37.3)	19 300 (12.5)	23 086 (15.0)
LGBTQIA+	3 708 (0.9)	686 (18.5)	806 (21.7)
Did not answer	8 055 (1.9)	1 300 (16.1)	1 508 (18.7)
**Disabilities**			
Without disability	111 051 (26.9)	10 508 (9.5)	15 166 (13.7)
With disability	43 685 (10.6)	9 160 (21.0)	10 519 (24.1)
Did not answer	258 721 (62.6)	43 494 (16.8)	51 939 (20.1)
**Highest education**			
College completed	182 345 (44.1)	21 811 (12.0)	29 372 (16.1)
High school completed	181 150 (43.8)	32 673 (18.0)	38 807 (21.4)
Less than high school	36 432 (8.8)	6 666 (18.3)	7 160 (19.7)
Did not answer	13 433 (3.2)	2 012 (15.0)	2 285 (17.0)
**Annual household income**			
≥200% FPL	185 292 (44.8)	22 168 (12.0)	30 285 (16.3)
<200% FPL	139 389 (33.7)	26 979 (19.4)	30 662 (22.0)
Did not answer	88 776 (21.5)	14 015 (15.8)	16 677 (18.8)
**Housing concern**			
Without housing concern	336 772 (81.5)	47 222 (14.0)	59 353 (17.6)
With housing concern	66 649 (16.1)	14 416 (21.6)	16 417 (24.6)
Did not answer	10 036 (2.4)	1 524 (15.2)	1 854 (18.5)
**Health insurance**			
Have health insurance	371 633 (89.9)	58 560 (15.8)	72 326 (19.5)
No health insurance	26 779 (6.5)	2 609 (9.7)	2 911 (10.9)
Did not answer	15 045 (3.6)	1 993 (13.2)	2 387 (15.9)
**Delayed care due to transportation**			
No	171 376 (41.4)	24 240 (14.1)	31 732 (18.5)
Yes	14 129 (3.4)	3 559 (25.2)	4 153 (29.4)
Did not answer	227 952 (55.1)	35 363 (15.5)	41 739 (18.3)
**Food insecurity**			
No food insecurity	100 995 (24.4)	12 532 (12.4)	16 738 (16.6)
Somewhat insecure	12 194 (2.9)	2 731 (22.4)	3 141 (25.8)
Insecure	3 548 (0.9)	895 (25.2)	998 (28.1)
Did not answer	296 720 (71.8)	47 004 (15.8)	56 747 (19.1)
**Neighbourhood safety**			
Safe	105 389 (25.5)	14 018 (13.3)	18 364 (17.4)
Not safe	9 642 (2.3)	1 809 (18.8)	2 125 (22)
Did not answer	298 426 (72.2)	47 335 (15.9)	57 135 (19.1)
**SDOH summary score**			
No risk	46 854 (11.3)	4 668 (10.0)	6 672 (14.2)
Have risk	266 997 (64.6)	47 604 (17.8)	55 852 (20.9)
Incomplete information	99 606 (24.1)	10 890 (10.9)	15 100 (15.2)

LGBTQIA+ refers to lesbian, gay, bisexual, transgender, queer, intersex, asexual, and others. Disabilities include blind, deaf, difficulty dressing/bathing, difficulty walking/climbing, difficulty running errands alone and difficulty concentrating. FPL stands for Federal Poverty Level 2023 which indicates the minimum amount of annual income that an individual/ family needs to pay for essentials in 2023. SDOH Summary Score is the sum of risk factors from all SDOH in this study. These included education attainment of high school and lower, < 200% FPL, with housing concerns, no health insurance, delayed care due to transportation, have food insecurity, and live in the unsafe neighbourhood. The score ranged from zero (0), no SDOH risk, to seven (7) have all risks. Participants were categorized into three groups: Have risk (score > 0), No risk (score = 0), and Incomplete information (missing any SDOH items and did not have any risk, if answered).

### Participants with mental health disorders

Among the cohort, 63,162 participants (15.3%) had MD, and 77,624 participants (18.8%) had AD. The majority of individuals diagnosed with MD were non-Hispanic White (56.8%), aged 50–64 years old (35.3%), and identified as female (66.3%). Most participants diagnosed with AD were non-Hispanic White (59.9%), aged 50–64 years old (32.6%) and identified as female (67.3%).

A greater number of participants with at least one disability had MD (21.0%) and/or AD (24.1%) compared to participants without disabilities (9.5% and 13.7%, respectively). Participants with less than high school education had a slightly higher rate of MD (18.3%) than those with college education (12.0%) and those with high school education (18.0%). Participants with an annual household income of <200% of the federal poverty level had a higher rate of MD (19.4%) and AD (22.0%) than those with an income of ≥200% of the federal poverty level (12.0% and 16.3%, respectively). Participants with housing concerns had a higher rate of MD (21.6%) and AD (24.6%) than those without housing concerns (14.0% and 17.6%, respectively). Also, participants who delayed getting medical care in the last 12 months had a higher rate of MD (25.2%) and AD (29.4%). A higher proportion of participants who experienced food insecurity were diagnosed with MD (25.2%) and AD (28.1%) compared to participants without food insecurity (12.4% and 16.6%, respectively). Similarly, participants who felt unsafe in their neighbourhood had a higher rate of MD (18.8%) and AD (22.0%) compared to participants who felt safe in their neighbourhood (13.3% and 17.4%, respectively). Interestingly, those who had health insurance reported a higher rate of MD (15.8%) and/or AD (19.5%) than participants without health insurance (9.7% and 10.9%, respectively) (Table 1). Characteristics of participants with MD and/or AD are shown in **Table S3**.

### *The associations between SDOH and the odds of having MD and* AD

After adjusting for covariates, both high school graduates (aOR, 1.38; 95% CI, 1.34–1.41 for MD and aOR, 1.31; 95% CI, 1.28–1.35 for AD) and those with less than a high school education (aOR, 1.43; 95% CI, 1.36–1.50 for MD and aOR, 1.29; 95% CI, 1.23–1.35 for AD) had higher odds of MD and AD than college graduates. Both earning <200% of the federal poverty level (aOR, 1.51; 95% CI, 1.46–1.55 for MD and aOR, 1.36; 95% CI, 1.32–1.40 for AD) and housing concern (aOR, 1.44; 95% CI, 1.39–1.49 for MD and aOR, 1.39; 95% CI, 1.35–1.43 for AD) were associated with higher odds of MD and AD than their counterparts. Those who had to delay getting the care they needed due to transportation were more likely to have MD and AD (aOR, 1.48; 95% CI, 1.39–1.58 for MD and aOR, 1.39; 95% CI, 1.31–1.47 for AD). However, participants without health insurance were less likely to have MD and AD (aOR, 0.48; 95% CI, 0.46–0.51 for MD and aOR, 0.44; 95% CI, 0.42–0.47 for AD). Food insecurity significantly increased the likelihood of MD (somewhat insecure: aOR, 1.38; 95% CI, 1.29–1.48 for MD and aOR, 1.20; 95% CI, 1.20–1.36 for AD, insecure: aOR, 1.29; 95% CI, 1.14–1.46 for MD and aOR, 1.28; 95% CI, 1.07–1.34 for AD) when compared to participants without food insecurity. Those who live in an unsafe neighbourhood also had higher odds of MD and AD (aOR, 1.18; 95% CI, 1.09–1.28 and aOR, 1.11; 95% CI, 1.03–1.19, respectively).

Finally, participants who had at least one SDOH risk factor had higher odds of both MD and AD compared to participants without any SDOH risk (aOR, 1.97; 95% CI, 1.89–2.06 and aOR, 1.69; 95% CI, 1.63–1.75, respectively), as shown in Table 2 **and Table S4**.

**Table 2.** Associations between social determinants of health (SDOH) and major depression and anxiety disorder in U.S. populations 2017–2022

	Major depression (*n* = 126 324)		Anxiety disorder (*n* = 155 248)	
Social determinants of health (SDOH)	Crude OR	Adj. OR	Crude OR	Adj. OR
**Highest education**				
College completed	Reference	Reference	Reference	Reference
High school completed	1.61 (1.57–1.65)	1.38 (1.34–1.41)	1.42 (1.39–1.45)	1.31 (1.28–1.35)
Less than high school	1.66 (1.59–1.72)	1.43 (1.36–1.50)	1.30 (1.25–1.35)	1.29 (1.23–1.35)
Did not answer	1.29 (1.21–1.37)	1.22 (1.13–1.32)	1.10 (1.03–1.16)	1.13 (1.05 −1.22)
**Annual household income**				
≥200% FPL	Reference	Reference	Reference	Reference
<200% FPL	1.74 (1.70–1.79)	1.51 (1.46–1.55)	1.44 (1.41–1.48)	1.36 (1.32–1.40)
Did not answer	1.33 (1.29–1.36)	1.26 (1.22–1.31)	1.18 (1.15–1.21)	1.26 (1.22–1.30)
**Housing concern**				
Without housing concern	Reference	Reference	Reference	Reference
With housing concern	1.69 (1.65–1.74)	1.44 (1.39–1.49)	1.53 (1.49–1.57)	1.39 (1.35–1.43)
Did not answer	1.13 (1.05–1.22)	1.09 (1.00–1.20)	1.07 (1.00–1.14)	1.11 (1.02–1.21)
**Health insurance**				
Have health insurance	0.58 (0.55–0.61)	0.48 (0.46–0.51)	0.50 (0.48–0.52)	0.44 (0.42–0.47)
No health insurance	Reference	Reference	Reference	Reference
Did not answer	0.82 (0.55–0.61)	0.69 (0.64–0.75)	0.79 (0.75–0.83)	0.69 (0.65–0.74)
**Delayed care due to transportation**				
No	Reference	Reference	Reference	Reference
Yes	2.12 (2.00–2.25)	1.48 (1.39–1.58)	1.86 (1.76–1.96)	1.39 (1.31–1.47)
Did not answer	1.12 (1.09–1.14)	0.89 (0.87–0.92)	0.99 (0.97–1.01)	0.86 (0.83–0.88)
**Food insecurity**				
No food insecurity	Reference	Reference	Reference	Reference
Somewhat insecure	2.09 (1.96–2.23)	1.38 (1.29–1.48)	1.78 (1.67–1.88)	1.20 (1.20–1.36)
Insecure	2.41 (2.15–2.70)	1.29 (1.14–1.46)	2.02 (1.82–2.24)	1.28 (1.07–1.34)
Did not answer	1.33 (1.30–1.37)	0.99 (0.86–1.13)	1.21 (1.18–1.24)	1.01 (0.90–1.14)
**Neighbourhood safety**				
Safe	Reference	Reference	Reference	Reference
Not safe	1.55 (1.44–1.67)	1.18 (1.09–1.28)	1.34 (1.25–1.43)	1.11 (1.03–1.19)
Did not answer	1.24 (1.20–1.27)	1.23 (1.07–1.41)	1.14 (1.11–1.16)	1.19 (1.06–1.34)
**SDOH summary score**				
No risk	NA	Reference	NA	Reference
Have risk	NA	1.97 (1.89–2.06)	NA	1.69 (1.63–1.75)
Incomplete information	NA	1.10 (1.05–1.15)	NA	1.09 (1.05–1.14)

FPL stands for Federal Poverty Level 2023 which indicates the minimum amount of annual income that an individual/family needs to pay for essentials in 2023. SDOH Summary Score is the sum of risk factors from all SDOH in this study. These included education attainment of high school and lower, < 200% FPL, with housing concerns, no health insurance, delayed care due to transportation, have food insecurity, and live in the unsafe neighbourhood. The score ranged from zero (0), no SDOH risk, to seven (7) have all risks. Participants were categorized into three groups: Have risk (score > 0), No risk (score = 0), and Incomplete information (missing any SDOH items and did not have any risk, if answered). The regression model was adjusted for race/ethnicity, age at consent, sexual orientation, and disabilities.

### The associations between covariates and mental health disorders

Higher odds of having MD and AD were also observed in under-represented groups including LGBTQIA+ (aOR, 1.53; 95% CI, 1.36–1.72 and aOR, 1.38; 95% CI, 1.24–1.54, respectively). Those with disabilities were more likely to have MD and AD (aOR, 1.96; 95% CI, 1.88–2.05 and aOR, 1.68; 95% CI, 1.62–1.75, respectively). The odds of having MD and AD were higher among those aged 35 years old or older. Nonetheless, race/ethnicity other than non-Hispanic White had lower odds of having MD, but had higher odds of having AD, as shown in **Table S4**.

## Discussion

We found strong evidence that SDOH across all five domains are associated with mental health disorders, except for health insurance coverage. For the education access and quality domain, several studies have shown that a higher level of educational attainment was associated with lower levels of anxiety and depression (Patria, 2022). Our results confirmed this association, as participants who graduated less than high school were 43% and 29% more likely to have MD and AD, respectively. However, a study argued that the relationship between the level of education and depression may be unique to countries (Patel *et al.*, 2018). For the economic stability domain, low levels of household income are associated with lifetime mental health disorders, and a reduction in household income is associated with an increased risk for mental health disorders (Melita *et al.*, 2021; Patel *et al.*, 2018; Sareen *et al.*, 2011). Our results also showed that people with income <200% of the federal poverty level were 51% and 36% more likely to have MD and AD, respectively.

Furthermore, housing instability has been linked to mental health disorders (Tsai, 2015; Tsai and Huang, 2019). This aligned with our results, which showed that people who were concerned about their housing situation were at least 39% more likely to have MD and AD. For the healthcare access and quality domain, the lack of transportation is a significant stressor that adversely affects mental health (Jahangeer *et al.*, 2021). This is also shown in our results by 48% and 39% increases in the odds of having MD and AD, respectively, in participants who have delayed getting the medical care they needed due to a lack of transportation. In contrast, our results showed that having no health insurance decreased the chance of having MD and AD significantly. This is contradicted by a recent study of 17,284 people which estimated the odds of having depression to be 71% higher for those who were uninsured. However, 7.2% of their samples had depression (Hughes and Hughes, 2022). For our study, the ratio of case-to-control was 1:1. This might represent the importance of using the optimum case-to-control ratio. On the other hand, our results may reflect the reverse causality of a higher rate of receiving a diagnosis for MD or AD when having health insurance as 89.9% of participants in the All of Us research programme were insured. Future studies with a longitudinal design should further examine the association between health insurance and mental health disorders.

For the neighbourhood and built environment domain, neighbourhood safety contributed to psychological distress (Booth *et al.*, 2012). Our study showed increases of 18% and 11% in the likelihood of having MD and AD, respectively, in people who lived in unsafe neighbourhoods. People who live in neighbourhoods with high rates of crime must cope with anxiety over their safety. This potent source of stress can lead to depression (Rosenbaum and Harris, 2001).

Longitudinal studies showed that adverse SDOH, including unemployment, family poverty, and food insecurity, led to higher risks of depression and anxiety (Alegría *et al.*, 2022; Zhou *et al.*, 2023). Also, supportive SDOH, including financial help and help with transportation, was associated with less depressive symptoms (Broadhead *et al.*, 1988; Brown *et al.*, 2012). Therefore, we might be able to estimate people’s risks of developing mental health disorders by evaluating SDOH. We created an SDOH summary score, which summarized the effects of SDOH risk factors from all five domains of SDOH to examine the collective effects of SDOH on mental health disorders. The summary score showed significant correlations with MD and AD. Participants who had at least one SDOH risk factor were 97% and 69% more likely to have MD and AD, respectively, than participants without any risk factor. As the importance of SDOH is increasingly recognized in clinical settings (Health Partners Plans, 2022; O’Gurek and Carla, 2018), our SDOH summary score might offer a quick assessment of social risks across all domains.

However, the cross-sectional design of our study does not allow for the establishment of the exposure variables before the outcome variable. A longitudinal study evaluating whether the SDOH summary score could predict the development of MD and AD needs to be done. Second, a high rate of missing data on some SDOH, including food insecurity and neighbourhood safety in which 71.8% and 72.2% of all participants did not provide this information, respectively, may cause an underestimation of the prevalence and reflect the data quality of the All of Us research programme. Third, the precision of categorizing participants into <200% of the federal poverty level could be improved by collecting income information in values, instead of intervals. Fourth, more SDOH risk factors should be included in the SDOH summary score. These include bullying experiences in social and community contexts domain (Ye *et al.*, 2023), and air and water quality in neighbourhood and built environments (MohanKumar *et al.*, 2008; Szyszkowicz *et al.*, 2009). Fifth, as this study focuses solely on data available on the All of Us research programme, future studies should reassess SDOH and mental health using other SDOH instruments (Moen *et al.*, 2020). However, to our knowledge, data in the All of Us research programme offers a more accurate representation of the U.S. population compared to other databases, due to its extensive inclusion of data from under-represented groups. Moreover, the research programme includes the largest dataset on SDOH, significantly enhancing the accuracy of our comprehensive study across all SDOH domains.

In conclusion, we found that five domains of SDOH were significantly associated with MD and AD occurrences. Almost all SDOH significantly increased the odds of having these mental health disorders, except for health insurance coverage. Our SDOH summary score also showed significant associations with mental health disorders which indicate a possible use of the summary score as a measure of risk of developing mental disorders.

## Supporting information

Tanarsuwongkul et al. supplementary material Tanarsuwongkul et al. supplementary material

## Data Availability

Data in the *All of Us* research program are released by the National Institutes of Health for the purpose of scientific research (https://www.researchallofus.org/). Registered researchers can access data and tools to conduct health research and improve the understanding of health and disease.

## Supplementary material

The supplementary material for this article can be found at https://doi.org/10.1017/S2045796024000866.
