# Supplementary material for: Associations between social determinants of health and mental health disorders among U.S. population: a cross-sectional study
Source: Epidemiol Psychiatr Sci. 2025 Jan 15;34:e4. doi: 10.1017/S2045796024000866 (PMC11735116; doi:10.1017/S2045796024000866)
Supplement: Tanarsuwongkul et al. supplementary material [file S2045796024000866sup001.docx]

**Supplementary Information**

*All of Us survey data handling*

For highest education attainment, participants were asked “What is the highest grade or year of school you completed?” in The Basics survey. Results include “Never Attended”, “One Through Four”, “Five Through Eight”, “Nine Through Eleven” (which categorized into “Less than high school”), “Twelve Or GED”, “College One to Three” (which categorized into “High school completed”), “College Graduate”, “Advanced Degree” (which categorized into “College completed”), “Prefer Not To Answer” and “Skip” (which categorized into “Did not answer”).

For annual household income, participants were asked “What is your annual household income from all sources?” in The Basics survey. Results include “less 10k”, “10k 25k”, “25k 35k”, “35k 50k”, “50k 75k”, “75k 100k”, “100k 150k”, “150k 200k”, “more 200k”, “Prefer Not To Answer” and “Skip”. To accurately categorize participants into “≥ 200% Federal Poverty Level” or “< 200% Federal Poverty Level”, we factor in the number of people in that household by using the following question: “Not including yourself, how many other people live at home with you?”. Responses include “zero” to “ten”, “11 or more household members”, “Response removed due to invalid value” and “Skip”. Participants who did not answer either of those questions and the response was removed were categorized into “Did not answer”. Other participants were categorized based on 200% of Federal Poverty Level (FPL) 2023 (ASPE, 2023).

For health insurance coverage, participants were asked “Are you covered by health insurance or some other kind of health care plan?” in The Basics survey. Results include “Yes” (“Have health insurance”), “No” (“No health insurance”), “Prefer Not To Answer”, “Dont Know” and “Skip”. The last three answers were combined into “Did not answer”.

For housing concern in the last 6 month, participants answered “Yes” or “No” to the question “In the past 6 months, have you been worried or concerned about NOT having a place to live?” in The Basics survey. These responses were categorized into “With housing concern” or “Without housing concern”, respectively. Participants who skipped this question were in “Did not answer” group.

For delayed care due to transportation, participants were asked “There are many reasons people delay getting medical care. Have you delayed getting care for any of the following reasons in the past 12 months? Didn't have transportation” in Healthcare Access & Utilization survey. Results include “Yes”, “No”, “Don’t Know” and “Skip”. The latter two results are combined in “Did not answer”.

For food insecurity level in the past 12 months, two questions provided in Social Determinants of Health survey were used: “Within the past 12 months, were you worried whether your food would run out before you got money to buy more?” and “Within the past 12 months, were you worried whether the food you had bought just didn't last and you didn't have money to get more?”. Responses for both questions include “Never true”, “Sometimes true” and “Often true”. These responses were assigned a score of zero to two, respectively. Then, a level of food insecurity was created for each participant by combining scores from both questions (level zero to four). Participants who answered “Never true” to both questions (score of zero) were categorized into “No food insecurity”. Participants who answered “Often true” to at least one question (score of three or four) were categorized into “Insecure”. If participants skipped both questions, they would be categorized into “Did not answer”. Other participants are in “Somewhat insecure” group.

For neighborhood safety, participants were asked “How much you agree or disagree that your neighborhood is safe?” in the Social Determinants of Health survey. “Safe” group includes “Strongly agree” and “Agree” while “Unsafe” includes "Strongly disagree” and “Disagree”. Participants who skipped this question were put in “Did not answer”.

**Supplementary Table 1. Codes used to ascertain participants with mental health disorders**

| **Mental Health Disorders** | **Observational Medical Outcomes Partnership code** | **SNOMED** |
| --- | --- | --- |
| Major depression | 4152280, 4282316, 4176002 | 370143000, 66344007, 42810003 |
| Anxiety disorder | 442077 | 197480006 |

**Supplementary Table 2. Scoring system for social determinant of health (SDOH) summary score**

| **Social determinant of health (SDOH)** | **Score** | **Domain** |
| --- | --- | --- |
| **Highest education** |  | Education access and quality |
| College completed | 0 |  |
| High school completed | 0.5 |  |
| Less than high school | 1 |  |
| Did not answer |  |  |
| **Annual household income** |  | Economic stability |
| ≥ 200% FPL | 0 |  |
| < 200% FPL | 1 |  |
| Did not answer |  |  |
| **Housing concern** |  |  |
| Without housing concern | 0 |  |
| With housing concern | 1 |  |
| Did not answer |  |  |
| **Health Insurance** |  | Health care access and quality |
| No health insurance | 1 |  |
| Have health insurance | 0 |  |
| Did not answer |  |  |
| **Delayed Care Due to Transportation** |  |  |
| No | 0 |  |
| Yes | 1 |  |
| Did not answer |  |  |
| **Food insecurity** |  | Social and community contexts |
| No food insecurity | 0 |  |
| Somewhat insecure | 1 |  |
| Insecure | 1 |  |
| Did not answer |  |  |
| **Neighborhood safety** |  | Neighborhood and built environment |
| Safe | 0 |  |
| Unsafe | 1 |  |
| Did not answer |  |  |
| **Summary score** |  |  |
| Have risk (score ≥ 1) |  |  |
| No risk (score = 0) |  |  |
| Incomplete information |  |  |

**Supplementary Table 3. Characteristics of participants with Major Depression and Anxiety Disorder**

| **Characteristics** | **All of Us participants**  **n (%)** | **Major depression**  **n (%)** | **Anxiety Disorder**  **n (%)** |
| --- | --- | --- | --- |
| **Total** | 413 457 (100.0) | 63 162 (15.3) | 77 624 (18.8) |
| **Race/Ethnicity** |  |  |  |
| Hispanic/Latino/a/x | 74 114 (17.9) | 11 029 (17.5) | 13 264 (17.1) |
| Non-Hispanic White | 222 646 (53.8) | 35 847 (56.8) | 46 479 (59.9) |
| Non-Hispanic Black | 77 069 (18.6) | 11 487 (18.2) | 11 764 (15.2) |
| Non-Hispanic Asian | 13 838 (3.3) | 776 (1.2) | 1 125 (1.4) |
| Non-Hispanic, >1 races | 6 836 (1.7) | 1 030 (1.6) | 1 302 (1.7) |
| Other | 7 262 (1.8) | 1 091 (1.7) | 1 398 (1.8) |
| Did not answer | 11 692 (2.8) | 1 902 (3.0) | 2 292 (3.0) |
| **Age at Consent** |  |  |  |
| 18 - 34 y | 99 133 (24.0) | 11 740 (18.6) | 16 674 (21.5) |
| 35 - 49 y | 94 319 (22.8) | 15 046 (23.8) | 19 381 (25.0) |
| 50 - 64 y | 126 638 (30.6) | 22 277 (35.3) | 25 268 (32.6) |
| ≥ 65 y | 93 365 (22.6) | 14 099 (22.3) | 16 301 (21.0) |
| **Sexual Orientation** |  |  |  |
| Female | 247 453 (59.8) | 41 876 (66.3) | 52 224 (67.3) |
| Male | 154 241 (37.3) | 19 300 (30.6) | 23 086 (29.7) |
| LGBTQIA+ | 3 708 (0.9) | 686 (1.1) | 806 (1.0) |
| Did not answer | 8 055 (1.9) | 1 300 (2.1) | 1 508 (1.9) |
| **Disabilities** |  |  |  |
| Without disability | 111 051 (26.9) | 10 508 (16.6) | 15 166 (19.5) |
| With disability | 43 685 (10.6) | 9 160 (14.5) | 10 519 (13.6) |
| Did not answer | 258 721 (62.6) | 43 494 (68.9) | 51 939 (66.9) |
| **Highest Education** |  |  |  |
| College completed | 182 345 (44.1) | 21 811 (34.5) | 29 372 (37.8) |
| High school completed | 181 150 (43.8) | 32 673 (51.7) | 38 807 (50.0) |
| Less than high school | 36 432 (8.8) | 6 666 (10.6) | 7 160 (9.2) |
| Did not answer | 13 433 (3.2) | 2 012 (3.2) | 2 285 (2.9) |
| **Annual Household Income** | |  |  |
| ≥ 200% FPL | 185 292 (44.8) | 22 168 (35.1) | 30 285 (39.0) |
| < 200% FPL | 139 389 (33.7) | 26 979 (42.7) | 30 662 (39.5) |
| Did not answer | 88 776 (21.5) | 14 015 (22.2) | 16 677 (21.5) |
| **Housing Concern** |  |  |  |
| Without housing concern | 336 772 (81.5) | 47 222 (74.8) | 59 353 (76.5) |
| With housing concern | 66 649 (16.1) | 14 416 (22.8) | 16 417 (21.1) |
| Did not answer | 10 036 (2.4) | 1 524 (2.4) | 1 854 (2.4) |
| **Health Insurance** |  |  |  |
| Without health insurance | 26 779 (6.5) | 2 609 (4.1) | 2 911 (3.8) |
| With health insurance | 371 633 (89.9) | 58 560 (92.7) | 72 326 (93.2) |
| Did not answer | 15 045 (3.6) | 1 993 (3.2) | 2 387 (3.1) |

**Supplementary Table 3. Characteristics of participants with Major Depression and Anxiety Disorder (cont)**

| **Characteristics** | **All of Us participants**  **n (%)** | **Major depression**  **n (%)** | **Anxiety Disorder**  **n (%)** |  |
| --- | --- | --- | --- | --- |
| **Delayed Care Due to Transportation** | |  |  |  |
| No | 171 376 (41.4) | 24 240 (38.4) | 31 732 (40.9) |  |
| Yes | 14 129 (3.4) | 3 559 (5.6) | 4 153 (5.4) |  |
| Did not answer | 227 952 (55.1) | 35 363 (56.0) | 41 739 (53.8) |  |
| **Food Insecurity** |  |  |  |  |
| No food insecurity | 100 995 (24.4) | 12 532 (19.8) | 16 738 (21.6) |  |
| Somewhat insecure | 12 194 (2.9) | 2 731 (4.3) | 3 141 (4.0) |  |
| Insecure | 3 548 (0.9) | 895 (1.4) | 998 (1.3) |  |
| Did not answer | 296 720 (71.8) | 47 004 (74.4) | 56 747 (73.1) |  |
| **Neighborhood Safety** |  |  |  |  |
| Safe | 105 389 (25.5) | 14 018 (22.2) | 18 364 (23.7) |  |
| Not safe | 9 642 (2.3) | 1 809 (2.9) | 2 125 (2.7) |  |
| Did not answer | 298 426 (72.2) | 47 335 (74.9) | 57 135 (73.6) |  |
| **SDOH summary score** |  |  |  |  |
| No risk | 46 854 (11.3) | 4 668 (7.4) | 6 672 (8.6) |  |
| Have risk | 266 997 (64.6) | 47 604 (75.4) | 55 852 (72.0) |  |
| Incomplete information | 99 606 (24.1) | 10 890 (17.2) | 15 100 (19.5) |  |

LGBTQIA+ refers to lesbian, gay, bisexual, transgender, queer, intersex, asexual, and others. Disabilities include blind, deaf, difficulty dressing/bathing, difficulty walking/climbing, difficulty running errands alone and difficulty concentrating. FPL stands for Federal Poverty Level 2023 which indicates the minimum amount of annual income that an individual/ family needs to pay for essentials in 2023. SDOH Summary Score is the sum of risk factors from all SDOH in this study. These included education attainment of high school and lower, < 200% FPL, with housing concerns, no health insurance, delayed care due to transportation, have food insecurity, and live in the unsafe neighborhood. The score ranged from zero (0), no SDOH risk, to seven (7) have all risks. Participants were categorized into three groups: Have risk (score > 0), No risk (score = 0), and Incomplete information (missing any SDOH items and did not have any risk, if answered).

**Supplementary Table 4. Associations between covariates and Major Depression and Anxiety Disorder in U.S. populations 2017 – 2022**

| **Social Determinants of Health (SDOH)** | **Major Depression (n = 126 324)** | | **Anxiety Disorder (n = 155 248)** | |
| --- | --- | --- | --- | --- |
|  | **Crude OR** | **Adj. OR** | **Crude OR** | **Adj. OR** |
| **Race/Ethnicity** |  |  |  |  |
| Hispanic | 0.90 (0.87 - 0.93) | 0.71 (0.68 - 0.73) | 0.82 (0.80 - 0.85) | 0.67 (0.65 - 0.69) |
| Non-Hispanic White | Reference | Reference | Reference | Reference |
| Non-Hispanic Black | 0.91 (0.88 - 0.93) | 0.66 (0.64 - 0.68) | 0.69 (0.67 - 0.71) | 0.53 (0.51 - 0.55) |
| Non-Hispanic Asian | 0.32 (0.29 - 0.35) | 0.38 (0.35 - 0.42) | 0.34 (0.32 - 0.37) | 0.38 (0.35 - 0.41) |
| Non-Hispanic. >1 races | 0.94 (0.87 - 1.03) | 0.89 (0.81 - 0.97) | 0.87 (0.80 - 0.94) | 0.78 (0.72 - 0.84) |
| Other | 0.90 (0.83 - 0.98) | 0.80 (0.73 - 0.87) | 0.93 (0.87 - 1.01) | 0.84 (0.77 - 0.91) |
| Did not answer | 1.01 (0.94 - 1.07) | 0.91 (0.84 - 0.99) | 0.94 (0.88 - 1.00) | 0.91 (0.84 - 0.98) |
| **Age at Consent** |  |  |  |  |
| 18 - 34 y | Reference | Reference | Reference | Reference |
| 35 - 49 y | 1.41 (1.37 - 1.46) | 1.43 (1.38 - 1.48) | 1.28 (1.24 - 1.32) | 1.30 (1.26 - 1.33) |
| 50 - 64 y | 1.58 (1.54 - 1.63) | 1.62 (1.56 - 1.67) | 1.25 (1.22 - 1.29) | 1.27 (1.23 - 1.30) |
| ≥ 65 y | 1.31 (1.27 - 1.35) | 1.48 (1.42 - 1.53) | 1.05 (1.01 - 1.08) | 1.09 (1.05 - 1.12) |
| **Sexual Orientation** |  |  |  |  |
| Female | 1.42 (1.38 - 1.45) | 1.46 (1.42 - 1.49) | 1.53 (1.49 - 1.56) | 1.52 (1.49 - 1.56) |
| Male | Reference | Reference | Reference | Reference |
| LGBTQIA+ | 1.54 (1.38 - 1.72) | 1.53 (1.36 - 1.72) | 1.60 (1.44 - 1.77) | 1.38 (1.24 - 1.54) |
| Did not answer | 1.40 (1.29 - 1.52) | 1.37 (1.24 - 1.52) | 1.35 (1.25 - 1.45) | 1.31 (1.19 - 1.44) |
| **Disability** |  |  |  |  |
| Without disability | Reference | Reference | Reference | Reference |
| With disability | 2.53 (2.43 - 2.63) | 1.96 (1.88 - 2.05) | 2.00 (1.93 - 2.07) | 1.68 (1.62 - 1.75) |
| Did not answer | 1.93 (1.88 - 1.98) | 1.72 (1.67 - 1.77) | 1.60 (1.56 - 1.63) | 1.51 (1.47- 1.55) |
